# Small-Molecule Lysophosphatidic Acid Receptor 5 (LPAR5) Antagonists: Versatile Pharmacological Tools to Regulate Inflammatory Signaling in BV-2 Microglia Cells

**DOI:** 10.3389/fncel.2019.00531

**Published:** 2019-11-29

**Authors:** Ioanna Plastira, Lisha Joshi, Eva Bernhart, Jens Schoene, Edgar Specker, Marc Nazare, Wolfgang Sattler

**Affiliations:** ^1^Gottfried Schatz Research Center, Molecular Biology and Biochemistry, Medical University of Graz, Graz, Austria; ^2^Leibniz-Forschungsinstitut für Molekulare Pharmakologie (FMP), Berlin, Germany; ^3^Berlin Institute of Health (BIH), Charite & MDC, Berlin, Germany; ^4^Center for Explorative Lipidomics, BioTechMed-Graz, Graz, Austria

**Keywords:** LPA5, microglia, chemokines, cytokines, neurotoxicity, transcription factors

## Abstract

Lysophosphatidic acid (LPA) species in the extracellular environment induce downstream signaling *via* six different G protein-coupled receptors (LPAR1–6). These signaling cascades are essential for normal brain development and function of the nervous system. However, in response to acute or chronic central nervous system (CNS) damage, LPA levels increase and aberrant signaling events can counteract brain function. Under neuro-inflammatory conditions signaling along the LPA/LPAR5 axis induces a potentially neurotoxic microglia phenotype indicating the need for new pharmacological intervention strategies. Therefore, we compared the effects of two novel small-molecule LPAR5 antagonists on LPA-induced polarization parameters of the BV-2 microglia cell line. AS2717638 is a selective piperidine-based LPAR5 antagonist (IC_50_ 0.038 μM) while compound 3 is a diphenylpyrazole derivative with an IC_50_ concentration of 0.7 μM in BV-2 cells. Both antagonists compromised cell viability, however, at concentrations above their IC_50_ concentrations. Both inhibitors blunted LPA-induced phosphorylation of STAT1 and STAT3, p65, and c-Jun and consequently reduced the secretion of pro-inflammatory cyto-/chemokines (IL-6, TNFα, IL-1β, CXCL10, CXCL2, and CCL5) at non-toxic concentrations. Both compounds modulated the expression of intracellular (COX-2 and Arg1) and plasma membrane-located (CD40, CD86, and CD206) polarization markers yet only AS2717638 attenuated the neurotoxic potential of LPA-activated BV-2 cell-conditioned medium towards CATH.a neurons. Our findings from the present *in vitro* study suggest that the two LPAR5 antagonists represent valuable pharmacological tools to interfere with LPA-induced pro-inflammatory signaling cascades in microglia.

## Introduction

The brain is an immune-privileged organ (Ransohoff and Brown, 2012), yet the central nervous system (CNS) is able to mount a primary immune response since host defense mechanisms are operative in astrocytes and microglia. Microglia are the resident immune cells of CNS and able to detect subtle alterations of the finely tuned micromilieu in the CNS (Ransohoff and Perry, 2009; Ransohoff and Cardona, 2010; Garaschuk and Verkhratsky, 2019; Norris and Kipnis, 2019). Microglia cover the whole CNS parenchyma and originate from embryonic progenitors in the yolk sac (Ginhoux et al., 2010; Butovsky and Weiner, 2018). These cells are unevenly distributed in the brain acquiring different morphologies, ranging from small, round cells to those having multiple branched processes (Hammond et al., 2018). These cells are a self-renewing, long-lived *in vivo* population, not replaced by peripheral monocytes (Ginhoux and Prinz, 2015), with a critical role in both, the physiological and pathological brain (Salter and Stevens, 2017; Hammond et al., 2018; Smolders et al., 2019). In their “resting” state, microglia processes scan their environment and respond to danger signals (Nimmerjahn et al., 2005). They are equipped with a unique cluster of transcripts encoding proteins for sensing endogenous ligands, collectively termed the microglia *sensome* (Hickman et al., 2013).

Within the last years, great progress in understanding and analyzing differences in microglia responses under pathological conditions has been made (Colonna and Butovsky, 2017; Wolf et al., 2017). Microglia regulate numerous aspects of inflammation, such as regeneration, cytotoxicity, and immunosuppression depending on their different activation states (Du et al., 2016). During disease progression they appear to be highly heterogeneous in terms of neurotoxic/pro-inflammatory or neuroprotective/anti-inflammatory responses (Tang and Le, 2016). Distinct molecular signatures and different microglia sub-populations have been identified, revealing major spatial, temporal and gender differences (Grabert et al., 2016; Guneykaya et al., 2018; Masuda et al., 2019), as well as differences associated with aging and context of the neurodegenerative disease (Colonna and Butovsky, 2017; Hickman et al., 2018; Song and Colonna, 2018; Mukherjee et al., 2019). Recently, the application of powerful methodologies has revealed unique phenotypic signatures under both physiological and neurodegenerative settings (Tay et al., 2018; Böttcher et al., 2019; Hammond et al., 2019; Masuda et al., 2019).

The lysophosphatidic acid (LPA) family consists of small alkyl- or acyl-glycerophospholipids (molecular mass: 430–480 Da) that act as extracellular signaling molecules through at least six G protein-coupled receptors (GPCRs; Yung et al., 2014). There is a range of structurally related LPA species present in various biological systems (Aoki, 2004). An important aspect of LPA receptor biology is that different LPA species may activate different LPA receptor isoforms (Kano et al., 2008). There are two major synthetic pathways for LPA (Yung et al., 2014). In the first pathway, phospholipids (PLs) are converted to their corresponding lysophospholipids such as lyso-phosphatidylcholine, -serine, or -ethanolamine. This occurs *via* phosphatidylserine-specific phospholipase A1 (PS-PLA1) or secretory phospholipase A2 (sPLA2) activity. Lysophospholipids are then converted to LPA *via* head group hydrolysis by autotaxin (ATX). In a second synthetic route, phosphatidic acid (PA), produced from PLs through phospholipase D (PLD) activity or from diacylglycerol (DAG) through diacylglycerol kinase (DGK) activity, is subsequently converted to LPA by the actions of either PLA1 or PLA2 (Aoki et al., 2008). LPA acts through specific G protein-coupled LPA receptors (LPAR1-LPAR6) that mediate the diverse effects of these lysophospholipids (Yung et al., 2014).

Under physiological conditions, LPA-mediated signaling is essential for normal neurogenesis and function of the CNS. However, in response to injury LPA levels can increase in brain and CSF (Tigyi et al., 1995; Savaskan et al., 2007; Ma et al., 2010; Yung et al., 2011; Santos-Nogueira et al., 2015). Aberrant LPA signaling contributes to multiple disease states, including neuropathic pain, neurodegenerative, neurodevelopmental and neuropsychiatric disorders, cardiovascular disease, bone disorders, fibrosis, cancer, infertility, and obesity (Yung et al., 2014). Microglia express LPA receptors and are activated by LPA (Möller et al., 2001; Bernhart et al., 2010). In the murine BV-2 microglia cells, LPA activates Ca^2+^-dependent K^+^ currents resulting in membrane hyperpolarization (Schilling et al., 2002) and induces cell migration *via* Ca^2+^-activated K^+^ channels (Schilling et al., 2004). In addition, LPA controls microglial activation and energy homeostasis (Bernhart et al., 2010), modulates the oxidative stress response (Awada et al., 2012), regulates the induction of chronic pain (Sun et al., 2012), and interferes with pro-inflammatory cytokine production (Awada et al., 2014).

LPAR5 was identified through screening approaches directed towards the deorphanization of GPR92 (Kotarsky et al., 2006). Signaling through the G_12/13_ pathway induces neurite retraction, stress fiber formation, and receptor internalization *in vitro*, while activation of G_q_ increases intracellular calcium levels and induce cAMP accumulation (Lee et al., 2006). LPAR5 is expressed in various tissues both in humans and mice (Amisten et al., 2008; Lundequist and Boyce, 2011; Yung et al., 2014). In the CNS, LPAR5 was found in the early embryonic forebrain, midbrain, and hindbrain of Slc:ddY mice. This expression pattern becomes more ubiquitous from E9.5–E12.5, showing diffuse patterns in the developing brain and choroid plexus revealing a role for LPAR5 in brain development (Ohuchi et al., 2008). LPAR5 signaling contributes to nerve injury-triggered pain (Lin et al., 2012; Ueda et al., 2013) and multiple sclerosis-associated neuropathic pain (Tsukahara et al., 2018). Findings that LPAR5 is activated during nerve injury (but not under basal conditions) are consistent with the fact that LPA levels rise significantly in response to spinal cord injury (Ma et al., 2010; Santos-Nogueira et al., 2015). Activated microglia were also suggested to contribute to demyelination in the injured spinal cord (Santos-Nogueira et al., 2015).

Although LPAR5 signaling was mainly studied in the context of neuropathic pain, we could recently show that LPAR5 affects microglia biology and induces a distinct pro-inflammatory and migratory signature (Plastira et al., 2016, 2017). The identification of the LPA/LPAR5 axis as a signaling pathway contributing to the inflammatory response of microglia might foster deeper insights into LPA-mediated effects on the resident immune cells of the brain. Since the binding domain of LPAR5 represents an extracellular target, it is ideally suited for pharmacological intervention. Accordingly, LPAR5 specific antagonists were developed to modulate the LPA/LPAR5 axis and study its role in development and progression of (neuro-) inflammatory diseases (Kozian et al., 2012, 2016; Murai et al., 2017; Kawamoto et al., 2018). In the present study, we analyzed the potential of two structurally diverse non-lipid LPAR5 antagonists (compound 3 and AS2717638; Kozian et al., 2016; Murai et al., 2017) to interfere with LPA-induced inflammatory signaling cascades in BV-2 microglia cells.

## Materials and Methods

### Materials

Cell culture medium RPMI1640, fetal calf serum (FCS), antibiotics, culture reagents, and trypsin were from Invitrogen (Waltham, MA, USA). LPA (1-oleoyl-2-hydroxy-sn-glycero-3-phosphate; LPA18:1) was from Sigma-Aldrich (St. Louis, MO, USA). Antibodies against COX-2, Arginase-1 and the nonphosphorylated and phosphorylated p65-NFkB, c-Jun, STAT1, and STAT3 were from Cell Signaling (Beverly, MA, USA); PE-CD40, APC-CD86, PE-CD206 antibodies and their isotype controls were from Biolegend (San Diego, CA, USA). Monoclonal anti-mouse β-actin (clone AC-74) was from Sigma-Aldrich (St. Louis, MO, USA). The LPAR5 inhibitors, AS2717638 [6,7-Dimethoxy-2-(5-methyl-1,2-benzoxazol-3-yl)-4-(piperidin-1-ylcarbonyl)isoquinolin-1(2H)-one] and compound 3 [4-((2-((1-(2,4-dichlorophenyl)-4-methyl-5-(4-(trifluoromethyl)phenyl)-1H-spyrazol-3-yl)methoxy)-2-methylpropanamido)methyl)benzoic acid] were synthesized according to the published procedures (Kozian et al., 2016; Murai et al., 2017).

For AS2717638, mass analysis was performed with an Agilent Technologies 6230 Accurate Mass TOF LC/MS linked to Agilent Technologies HPLC 1260 Series using a Thermo Accuore RP-MS column (30 × 2.1 mm, 2.6 μm particle size; Eluent A: H_2_O with 0.1% formic acid Eluent B: MeCN with 0.1% formic acid; Gradient: 0.00 min 95% A, 0.2 min 95% A, 1.1 min 1% A, 2.5 min stop-time, 1.3 min post-time; Flow rate: 0.8 ml/min; UV-detection: 220 nm, 254 nm, and 300 nm). LC-MS: R_t_ = 1.873 min; HRMS (ESIpos): *m/z* [M + H]^+^ experimentally determined = 448.1454, calculated for C_25_H_25_N_3_O_5_ = 448.1467.

^1^H NMR (300 MHz, DMSO-*d*_6_) δ 7.80–7.75 (m, 2H), 7.64 (s, 1H), 7.62–7.56 (m, 2H), 6.95 (s, 1H), 3.94 (s, 3H), 3.93 (s, 3H), 3.75–3.52 (m, 2H), 3.52–3.36 (m, 2H), 2.44 (s, 3H), 1.68–1.54 (m, 4H), 1.54–1.43 (m, 2H). ^ 13^C NMR (75 MHz, DMSO) δ 164.7, 163.1, 159.5, 156.8, 154.6, 150.1, 134.3, 133.2, 130.0, 128.8, 122.9, 119.2, 117.7, 115.3, 110.6, 108.6, 105.2, 56.4, 56.3, 26.8, 24.4, 21.0.

SwissADME^1^ was used for drawing, chemical structure property prediction, and calculations (Daina et al., 2017).

### BV-2 Microglia Culture

The murine microglial cell line BV-2 was from Banca Biologica e Cell Factory (Genova, Italy). Cells were cultivated and maintained in RPMI1640 medium supplemented with 10% FCS, 100 U/ml penicillin, 100 mg/ml streptomycin, and 5 ml L-glutamine (200 mM) at 37°C in a humidified incubator under 5% CO_2_ and 95% air. When cells reached confluency, they were split into new flasks or they were plated accordingly for the experiments as described previously (Plastira et al., 2016).

### CATH.a Neurons Culture

The murine neuronal cell line CATH.a was from ATCC. Cells were grown and maintained in RPMI1640 medium supplemented with 10% horse serum, 5% FCS, 1% penicillin-streptomycin, 0.4% HEPES, and 0.2% sodium pyruvate at 37°C (5% CO_2_). When cells reached confluency, they were split into new flasks (subcultivation ratio of 1:4) using 0.12% trypsin without EDTA as described (Waltl et al., 2013).

### LPA Treatment

Cells were plated in 6 or 12-well plates and allowed to adhere for 2 days. Cells were always kept in serum-free medium overnight before incubation with LPA and LPA plus each inhibitor. Aqueous LPA stock solutions (5 mM) were stored at −70°C. Only freshly thawed stocks were used for the experiments.

### Inhibitor Treatments

AS2717638 and compound 3 were diluted in DMSO (stock concentration: 10 mM) and kept at −20°C. During the experiments, AS2717638 and compound 3 were used at a final concentration of 0.1 μM and 1 μM, respectively. The highest DMSO concentration was 0.01% (v/v) and used as vehicle control throughout the study.

### MTT Assay

The toxicity of the two inhibitors on BV-2 cells was assessed using the MTT assay. Cells were plated in 24-well plates and grown to confluency. The cells were incubated with MTT (1.2 mM; in serum-free medium) for 1 h, washed with PBS and cell lysis was performed with isopropanol/1 M HCl (25:1 v/v) on a rotary shaker at 1,200 rpm for 10 min. Finally the samples were diluted 1:5 and absorbance was measured at 570/650 nm on a Victor 1,420 multilabel counter (Wallac).

### Immunoblotting

After treatment with the experimental substances for the indicated time periods, BV-2 cells were washed twice with ice-cold PBS and lysed in RIPA buffer (50 mM Tris-HCl pH 7.4, 1% NP-40, 150 mM NaCl, 1 mM Na_3_VO_4_, 1 mM NaF, 1 mM EDTA) containing protease inhibitors (Sigma; aprotinin, leupeptin, pepstatin: 1 μg/ml each), 10 μM PMSF and phosphatase inhibitors cocktail (Thermo Scientific, Waltham, MA, USA). Protein content was determined using the BCA kit (Thermo Scientific) and BSA as standard. Protein samples (100 μg) were separated on 10% SDS-PAGE gels and transferred to polyvinylidene difluoride membranes. Membranes were blocked with 5% low-fat milk in Tris-buffered saline containing Tween 20 (TBST) for 2 h at RT and incubated with the primary antibodies overnight with gentle shaking at 4°C. After the removal of primary antibodies, the membranes were washed for 30 min in TBST and incubated for 2 h at RT with anti-rabbit (1:10,000) or anti-mouse (1:5,000) as secondary antibodies. Following three washes with TBST for 1 h, immunoreactive bands were visualized using ECL or ECL plus reagents and detected with a chemiluminescence detection system (ChemiDoc Bio-Rad, Berkeley, CA, USA). In some cases, the membranes were stripped using a stripping buffer (140 μl β-mercaptoethanol in 20 ml buffer 60 mM Tris/ 2% SDS, pH 6.8) under gentle shaking for 30 min at 50°C in a water bath, washed for 1 h in TBST, blocked with 5% low-fat milk in TBST for 1 h at room temperature and probed with the pan antibodies for p65-NF-kB, c-Jun, STAT1, and STAT3 as described (Plastira et al., 2017). Anti-β-actin (1:5,000) was used as loading controls.

### Flow Cytometry

Flow cytometry was used to quantitate the percentage of CD40, CD86, and CD206 positive microglia cells. BV-2 cells were seeded in triplicate onto 6-well at a density of 1 × 10^5^ cells per well. After 24 h serum-starvation, cells were incubated with vehicle control, LPA or LPA plus the antagonists for 12 and 24 h. Cells were then collected, blocked using the Ultra V blocker (Thermo Scientific) and incubated with PE anti-CD40, APC anti-CD86, or PE anti-CD206 antibody (1:50). After fixation, samples were measured using a Guava easyCyte 8 Millipore flow cytometer.

### ELISA

IL-1β, TNFα, IL-6, CCL5 (RANTES), CXCL2 (MIP-2), and CXCL10 (IP-10) concentrations in the cellular supernatants were quantitated using the murine ELISA development kits (Peprotech, NJ, USA; Plastira et al., 2016). Briefly, BV-2 cells were seeded in triplicate onto 12-well plates at a density of 5 × 10^4^ cells per well, serum-starved (o/n), and incubated in serum-free medium, containing LPA in the absence or presence of the antagonists for the indicated time periods. For each time point, the supernatants were collected and kept at −70°C until further use. The concentrations of the cytokines and chemokines were determined using the external standard curve.

#### LDH Assay

Lactate dehydrogenase is a soluble enzyme located in the cytosol and released into the culture medium upon cell lysis or damage. LDH activity can, therefore, be used as an indicator of membrane integrity and thus a measurement of cytotoxicity (Cayman Chemical, Ann Arbor, MI, USA). The assay was performed as previously described (Plastira et al., 2016). In brief, BV-2 cells were seeded in triplicate into 6-well plates at a density of 1 × 10^5^ cells per well, serum-starved overnight and incubated in serum-free medium, containing LPA in the absence or presence of the antagonists for the indicated time periods. For each time point, the supernatants were collected and kept at −70°C until further use.

CATH.a neurons were seeded in a 96-well plate at a concentration of 1 × 10^5^ cells per well, and following overnight serum-starvation, the cells were incubated in the presence of the above-mentioned supernatants. Three wells containing only medium without cells were used for background control. In order to measure maximum and spontaneous release, cells were incubated with 10% Triton X-100 and assay buffer, respectively. After 24 h, the plate was centrifuged at 1,300 rpm for 5 min. One-hundred microliter of the supernatants was transferred to a new 96-well plate and 100 μl of LDH reaction solution was added to each well. The plate was incubated at 37°C for 30 min and the absorbance at 490 nm was measured using a plate reader (Plastira et al., 2017).

#### Statistical Analysis

All experiments were performed using three replicates per experimental group and repeated three times (unless otherwise stated). Statistical analyses were performed using the GraphPad Prism version 6 for Mac (GraphPad Software, Inc., San Diego, CA, USA). Data obtained from independent measurements were analyzed by one-way ANOVA followed by Bonferroni’s *post hoc* test and presented as mean ± SD.

## Results

### AS2717638 and Compound 3 Inhibit LPA-Mediated Pro-inflammatory Transcription Factor Phosphorylation

**Figure 1:**
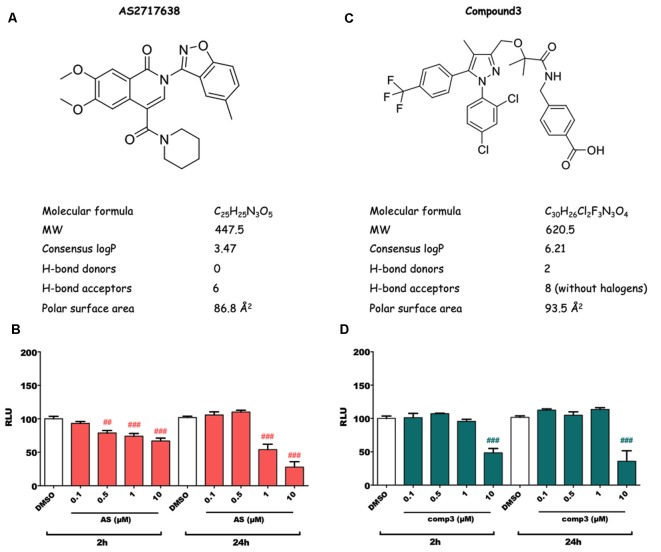
Structure, physicochemical properties and cytotoxic potential of AS2717638 and compound 3. Chemical structures of AS2717638 **(A)** and compound 3 **(C)**. SwissADME was used to calculate physicochemical properties relevant to the “*rule of five*” that predict drug-likeliness of a compound (Lipinski et al., 2001). Cytotoxicity was assessed using the MTT assay. BV-2 microglia cells were cultured in 6-well plates, serum-starved overnight (o/n) and incubated with the indicated concentrations of AS2717638 **(B)** and compound 3 **(D)** in DMSO for 2 and 24 h. DMSO was used as vehicle control. For convenience, the names of the inhibitors are presented as AS and comp3. Results from three independent experiments in triplicates are presented as mean ± SD (^##^*p* < 0.01, ^###^*p* < 0.001 compared to vehicle control; one-way ANOVA with Bonferroni correction).

Structures and physicochemical properties of AS2717638 and compound 3 are displayed in Figures 1A,C. As a first step in our study, we analyzed whether the two LPAR5 antagonists exhibit toxic effects in BV-2 microglia cells. Incubation with increasing concentrations of AS2717638 (0.1–10 μM) revealed that BV-2 cell viability was reduced between 10% and 30% after a 2 h incubation at concentrations ≥0.5 μM (Figure 1B). Therefore AS2717638 was used at 0.1 μM in all experiments. After a 24 h incubation, cell viability was decreased by 55 and 70% (1 and 10 μM, respectively; Figure 1B). In contrast, compound 3 reduced viability by 50 (2 h) and 60 (24 h) % only at the highest concentration (10 μM) used (Figure 1D). In the experiments described below, compound 3 was used at 1 μM, a concentration without detrimental effects on cell viability. At these concentrations, both inhibitors attenuated LPA-mediated morphological changes (cell perimeter and cellular surface area) of BV-2 cells (Supplementary Figure S1).

**Figure 2:**
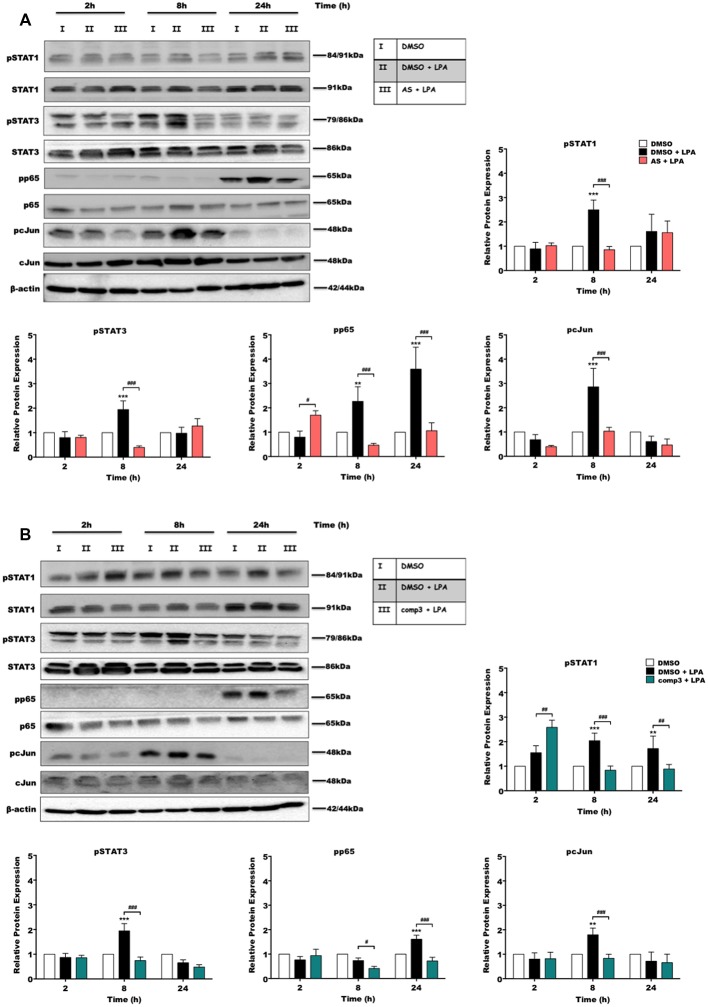
AS2717638 and compound 3 attenuate lysophosphatidic acid (LPA)-induced phosphorylation of pro-inflammatory transcription factors. BV-2 microglia cells were cultured in 6-well plates and serum-starved o/n. Cells were treated with DMSO, DMSO plus LPA (1 μM), and LPA (1 μM) in the presence of **(A)** AS2717638 (0.1 μM) or **(B)** compound 3 (1 μM) for the indicated time periods and cell protein lysates were collected. The phosphorylation states along with the total levels of STAT1, STAT3, p65-NF-kB, and c-Jun were detected using Western blotting. If two bands appeared for one protein [e.g., (p)STAT3] both bands were included in the densitometric evaluation. Protein/loading control ratios were normalized to the ratio of unstimulated microglia (mean value of DMSO controls was set to 1). One representative blot out of three separate experiments and the densitometric analysis of each protein expression from three independent experiments is presented (**p < 0.01; ***p < 0.001 compared to DMSO-treated cells; ^#^*p* < 0.05; ^##^*p* < 0.01; ^###^*p* < 0.001 each inhibitor compared to LPA-treated cells; one-way ANOVA with Bonferroni correction).

During previous experiments, we examined the effect of LPA on microglial inflammatory response and reported that LPAR5 controls the LPA induced pro-inflammatory phenotype in microglia cells (Plastira et al., 2017). In order to test the impact of AS2717638 and compound 3 on activation/phosphorylation of transcription factors in BV-2 cells in response to LPA (1 μM) treatment, we used immunoblot analysis. Cells were incubated in the presence or absence of each inhibitor for different time periods and the activation of transcription factors was analyzed. Results of these experiments indicated that both LPAR5 antagonists clearly suppressed LPA-induced phosphorylation of p65-NF-kB, c-Jun, STAT1, and STAT3 (Figure 2). AS2717638 used at 0.1 μM attenuated STAT1 and STAT3 phosphorylation back to or below baseline at the 8 h time point, and comparable results were obtained for pp65 and pcJun (Figure 2A). Also compound 3 (1 μM) decreased phosphorylation of all transcription factors at one or more time points (Figure 2B). The bar graphs represent densitometric analysis from three independent experiments.

### AS2717638 and Compound 3 Attenuate LPA-Induced Cyto-/Chemokine Secretion by BV-2 Microglia

**Figure 3:**
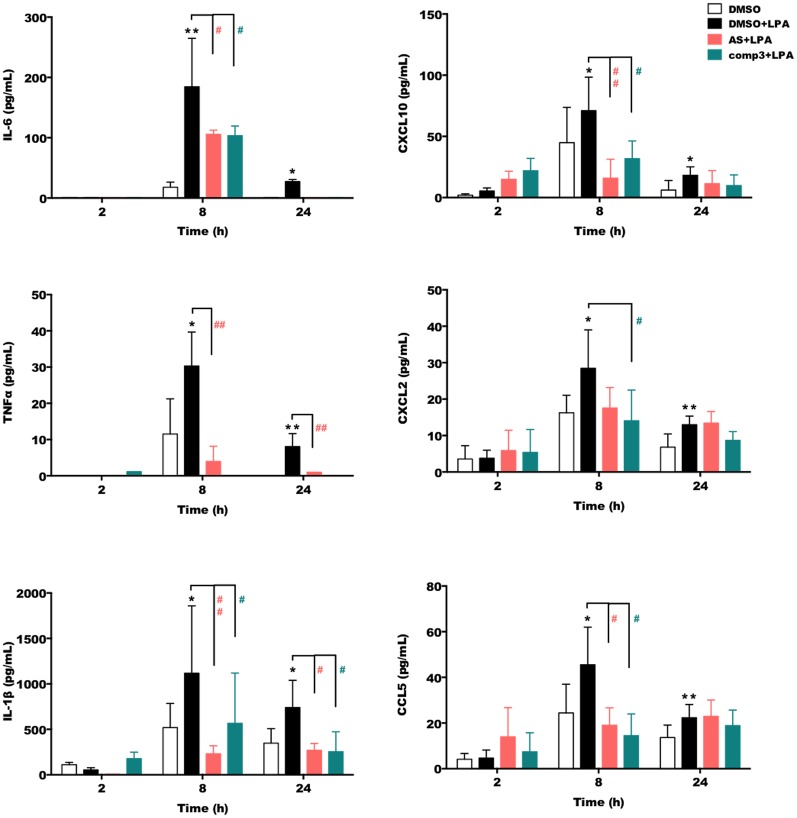
AS2717638 and compound 3 reduce LPA-mediated secretion of pro-inflammatory cytokines and chemokines. BV-2 microglia were cultured on 24-well plates and serum-starved o/n. The supernatants were collected after incubation with vehicle control (DMSO), DMSO plus LPA (1 μM) or LPA plus AS2717638 (0.1 μM) or compound 3 (1 μM) for 2, 8, and 24 h. ELISA was used to quantify the concentrations of IL-6, TNFα, IL-1β, CXCL10 (IP-10), CXCL2 (MIP-2), and CCL5 (RANTES). Results shown represent mean ± SD from three independent experiments performed in triplicate (*p < 0.05; **p < 0.01 compared to vehicle control; ^#^*p* < 0.05, ^##^*p* < 0.01; each inhibitor compared to LPA-treated cells; one-way ANOVA with Bonferroni correction). No bars = below detection limit.

We then quantitated the concentrations of secreted IL-6, TNFα, IL-1β, CXCL10, CXCL2, and CCL5 using ELISA kits (Figure 3). These experiments revealed that both, AS2717638 and compound 3 decreased secretion of IL-6, TNFα, IL-1β, CXCL10, CXCL2, and CCL5 almost back to base-line levels. For AS2717683 the decrease in CXCL2 concentrations was statistically not significant.

### LPA-Induced Pro-inflammatory Marker Expression Is Attenuated by AS2717638 and Compound 3

**Figure 4:**
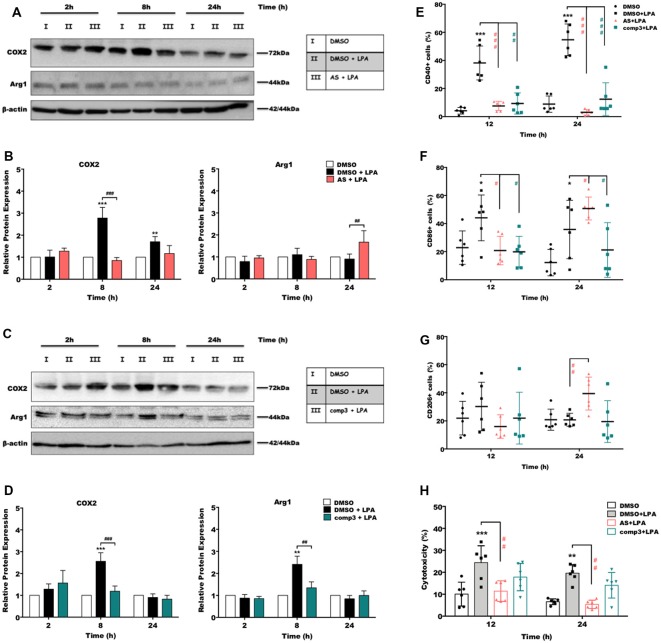
AS2717638 and compound 3 restore a neuroprotective microglial phenotype while only AS2717638 reduces neurotoxicity of microglia-conditioned medium. Serum-starved microglia cells were treated with DMSO, DMSO plus LPA (1 μM), and LPA plus AS2717638 (0.1 μM; **A**) or compound3 (1 μM; **C**) for 2, 8, and 24 h. Cell lysates were collected and the expression of COX-2 and Arg-1 was monitored by immunoblotting. One representative plot for each protein and the densitometric analysis (**B,D**; mean ± SD) from three independent experiments are presented (mean value of DMSO controls was set to 1). In a parallel experiment, serum-starved (o/n) BV-2 cells were cultivated in the presence of DMSO, DMSO plus LPA (1 μM) or LPA in the presence of AS2717638 (0.1 μM) or compound 3 (1 μM) for the indicated times. Cells were stained with PE-conjugated anti-CD40 **(E)**, APC-conjugated anti-CD86 **(F)** or PE-conjugated anti-CD206 **(G)** antibodies and analyzed using a Guava easyCyte 8 Millipore flow cytometer. Results are shown as mean values ± SD. **(H)** CATH.a neurons were incubated for 24 h with conditioned media collected from LPA-treated BV-2 cells in the presence or absence of AS2717638 (0.1 μM) or compound 3 (1 μM) for 12 and 24 h. The LDH levels were detected and cytotoxicity was calculated according to the manufacturer’s instructions (*p < 0.05; **p < 0.01; ***p < 0.001 compared to DMSO-treated cells; ^#^*p* < 0.05; ^##^*p* < 0.01; ^###^*p* < 0.001 each inhibitor compared to LPA-treated cells; one-way ANOVA with Bonferroni correction).

In the next set of experiments, we analyzed the impact of LPAR5 antagonism on the expression of pro- and anti-inflammatory markers using Western blotting and flow cytometry. Immunoblotting experiments revealed that treatment with AS2717638 (Figure 4A) or compound 3 (Figure 4C) significantly reduced LPA-dependent COX-2 expression yet only AS2717638 could increase the expression of Arg-1, a marker protein for the M2 microglia/macrophage phenotype. Densitometric evaluation of immunoreactive bands from three separate experiments is given in the bar graphs (Figures 4B,D). Using flow cytometry, we then analyzed the expression pattern of specific polarization surface markers in LPA-stimulated BV-2 cells in the absence or presence of the antagonists. As shown in Figures 4E,F, both antagonists abrogated LPA-mediated CD40 and CD86 (except AS2717638 at 24 h) upregulation, whereas CD206 levels were only upregulated by AS2717638 (Figure 4G). Scatterplots representative of these analyses are shown in Supplementary Figure S2.

### AS2717638, but Not Compound 3, Reduces Neurotoxic Properties of Conditioned Medium Collected From LPA-Stimulated BV-2 Cells

CATH.a neurons were incubated with the supernatants collected from LPA-treated (in the absence or presence of AS2717638 or compound 3) BV-2 cells. Neuronal cell death was quantified using an LDH activity kit (Plastira et al., 2017). BV-2 medium collected from LPA-stimulated cells induced a 2.5-fold increase in LDH activity in CATH.a cultures (Figure 4H, gray bars). In contrast, medium collected from LPA-activated microglia that were cultured in the presence of AS2717638 did not affect neuronal viability (red bars). Although compound 3 showed a tendency to decrease cytotoxic effects of microglia-conditioned medium these effects were statistically not significant (Figure 4H, green bars).

## Discussion

Depending on the disease context, extrinsic signals determine whether microglia acquire a beneficial or detrimental phenotype. Their critical role in CNS homeostasis makes these cells potential therapeutic targets, which necessitate a thorough understanding of different phenotypic subclasses, transcriptome profiles, and pathways that modulate their function.

In numerous pathological conditions, such as (neuro-) inflammation, brain injury, neuropathic pain and gliomas, LPA levels can increase and through downstream signaling counteract brain function. Aberrant ATX-LPA signaling has been implicated in several neurological disorders including neuropathic pain and schizophrenia (Yang et al., 2015). LPA levels were elevated in a controlled cortical impact mouse model of TBI (Crack et al., 2014; Eisenried et al., 2017), in Huntington’s disease mouse brains (Vodicka et al., 2015) and in glioblastoma multiforme (Tabuchi, 2015). Tissue distribution analyses revealed that ATX gene expression is induced in human AD frontal cortex and the TauP301L mouse model (Umemura et al., 2006). Altered expression levels and activity of ATX with accompanying alterations in LPA signaling have recently been implicated in the pathogenesis of AD (Ramesh et al., 2018).

Due to its importance in numerous diseases, ATX makes an attractive target for therapeutic intervention (Matralis et al., 2019). However, there may be therapeutic benefit by selectively targeting one or more LPA receptor subtypes (Herr et al., 2018). Unfortunately, up to date, only a few selective antagonists for LPAR5 have been reported (Kozian et al., 2012, 2016; Murai et al., 2017). In a previous study, using one of those specific LPAR5 inhibitors (TCLPA5), we unraveled that the LPA/LPAR5 axis controls the inflammatory and migratory response in microglia cells (Plastira et al., 2016). In the present study, we analyzed the potency of two newly developed small molecule LPAR5 antagonists (AS2717638 and compound 3) to interfere with this LPA-induced pro-inflammatory response. AS2717638 was successfully used in preclinical rodent models of neuropathic pain where the inhibitor showed high potency, selectivity, and CNS penetration with broad analgesic effects (Murai et al., 2017). Compound 3 is a diphenylpyrazole compound that was identified during a compound library screening approach (Kozian et al., 2016). Compound 3 is metabolically stable, has good absorption properties, and is bioavailable after oral administration in mice (Kozian et al., 2016). *In vitro*, compound 3 inhibits LPA-mediated activation (MCP-1 expression) of human mast cells or BV-2 microglia. However, and in contrast to AS2717638, compound 3 displays physicochemical properties that are predicted to have (according to Lipinski’s “rule of five”; Lipinski et al., 2001) poor oral absorption and/or distribution properties: the molecular weight is >500, logP is >5, and there are eight hydrogen-bond acceptors present in the compound (Figure 1C). On the other hand the polar surface area is <140 Å^2^, which could compensate the high molecular weight (Veber et al., 2002) and mediate the good oral bioavailability observed in C57BL/6 mice (Kozian et al., 2016).

A first important observation from our *in vitro* study is that AS2717638 can regulate the BV-2 response to LPA at a 10-fold lower concentration compared to compound 3 (0.1 μM vs. 1 μM). Although both compounds have the potential to decrease cell viability, the concentrations inducing detrimental effects were far higher than the reported IC_50_ values. These findings indicate an appropriate (*in vitro*) effect-to-toxicity window that is devoid of unspecific cellular responses. The inhibitory effects of the two LPAR5 antagonists were first assessed by analyzing their impact on the LPA-induced expression of different transcription factors. c-Jun is a component of AP-1 transcription factors and regulates the expression of many inflammatory and cytokine genes, which are involved in brain inflammation (Raivich, 2008). In addition, constitutive and inducible activation NF-kB in glial cells regulates inflammatory processes that exacerbate various diseases (Kaltschmidt and Kaltschmidt, 2009). Specifically in microglia, NF-kB regulated activation induced the release of pro-inflammatory cytokines and caused neurotoxicity (Block et al., 2007; Khasnavis et al., 2012; Yao et al., 2013; Frakes et al., 2014; Parisi et al., 2016). AS2717638 and compound 3 effectively inhibited phosphorylation of both c-Jun and p65 in LPA treated BV-2 cells at 8 and/or 24 h post-inhibitor addition indicating long-term regulation (Figures 2A,B). However, it should be noted that BV-2 cells used during the present study exhibit less pronounced induction of pro-inflammatory genes and lower cytokine secretion in response to LPS when compared to primary microglia (He et al., 2018), facts indicating potential limitations for the use of the BV-2 model.

In the CNS, STAT proteins are associated with development, hormone release, tumorigenesis, and inflammation (Nicolas et al., 2013). STATs are mediators of immunity and play important roles in inflammatory disease (O’Shea and Plenge, 2012). In brain tumors, STAT3 is highly upregulated (Gu et al., 2008; Chen et al., 2010). The role of STAT3 in brain inflammation is not entirely clear since both anti-inflammatory and pro-inflammatory mediators can activate it. In microglia, it was reported that the JAK2-STAT3 pathway induces pro-inflammatory responses (Huang et al., 2008; Yang et al., 2010). In contrast, the role of STAT1 is more clear-cut and usually promotes inflammation, expression of different cytokines, and production of NO and ROS (Delgado, 2003; Rezai-Zadeh et al., 2008; Herrera-Molina et al., 2012; Rauch et al., 2013). Different expression levels have been detected in glial cells (De-Fraja et al., 1998) and are associated with CNS pathological conditions such as brain inflammation (Hashioka et al., 2009), traumatic brain injury (Okada et al., 2006), and cerebral ischemia (Choi et al., 2005; Satriotomo et al., 2006). Both antagonists significantly decreased the activation of STAT3. Differences have been observed during STAT1 phosphorylation. Even though both, AS2717638 and compound 3 abrogated STAT1 activation at 8 h, AS2717638 lost its inhibitory potential at the 24 h time point.

Microglia activation is accompanied by directed migration to the site of injury and subsequent release of cytokines, chemokines, NO, or ROS, which can have beneficial or detrimental effects on bystander cells (Ransohoff and Perry, 2009). The time-dependent expression profiles of cyto-/chemokines are specific for a pro- or anti-inflammatory microglia phenotype (Chhor et al., 2013). In response to LPA, an upregulated expression of IL-6, TNFα, IL-1β, CXCL10, CXCL2, and CCL5 was observed, in line with reports for LPS-activated primary murine microglia (Chhor et al., 2013). In addition, IL-1β, IL-6, TNFα, and the chemokines CCL5, and CXCL2 are implicated as regulators of the inflammatory response in (animal) models of TBI (Gyoneva and Ransohoff, 2015) where LPA concentrations are elevated (Crack et al., 2014). AS2717638 and compound 3 significantly decreased LPA-induced expression of those cytokines and chemokines, at least at one time point. Differences were observed for CXCL2, where AS2717638 had no statistically significant inhibitory effect. Both inhibitors abrogated the expression of COX2, CD40 and CD86 (pro-inflammatory markers). This is in line with earlier findings where we could demonstrate that TCLPA5 (an LPAR5 inhibitor) attenuates ERK1/2 and JNK activation, which both couple to COX-2 expression (Plastira et al., 2017). We can currently not offer an explanation for Arg1 upregulation in response to LPA (Figure 4D). In contrast to compound 3 only AS2717638 upregulated the anti-inflammatory markers Arg1 and CD206 (Figures 4B,G).

Microglia-induced neurotoxicity (Biber et al., 2014) is mediated by the constant production of pro-inflammatory cytokines and chemokines, NO (Liu et al., 2002), and ROS (Hsieh and Yang, 2013). An increase in oxidative stress might have detrimental effects (e.g., cell membrane damage, oxidative modification of lipids, covalent modification of intracellular proteins, or reduced antioxidant capacity of neurons) thereby promoting disease progression (Valko et al., 2007; Hsieh and Yang, 2013). During the present study, we found that supernatants collected from LPA plus AS2717638-treated BV-2 cells ameliorated cytotoxicity towards CATH.a neurons. Although compound 3 treatment showed a tendency for decreased neurotoxicity these effects were statistically not significant.

In conclusion, both LPAR5 antagonists are promising candidates to pharmacologically modulate the pro-inflammatory LPA/LPAR5 axis in microglia since AS2717638 and compound 3 abrogated phosphorylation of pro-inflammatory transcription factors and reduced secretion of inflammatory cytokines and chemokines. Our results point towards the possibility that LPAR5 antagonists might be useful pharmacological compounds to dampen the neuro-inflammatory response of microglia in CNS diseases that associate with aberrant LPA production.

## Data Availability Statement

All datasets generated for this study are included in the article/Supplementary Material.

## Author Contributions

IP, EB, and WS designed the study, analyzed the data and wrote the manuscript. LJ performed the cytotoxicity and immunoblotting experiments. IP performed the immunoblotting, ELISA, flow cytometry and cytotoxicity experiments. JS, ES, and MN synthesized and analytically characterized inhibitors and analyzed data. WS supervised the study.

## Conflict of Interest

The authors declare that the research was conducted in the absence of any commercial or financial relationships that could be construed as a potential conflict of interest.

## Footnotes

^1^http://www.swissadme.ch/
