# Supplementary material for: Small-Molecule Lysophosphatidic Acid Receptor 5 (LPAR5) Antagonists: Versatile Pharmacological Tools to Regulate Inflammatory Signaling in BV-2 Microglia Cells
Source: Front Cell Neurosci. 2019 Nov 29;13:531. doi: 10.3389/fncel.2019.00531 (PMC6897279; doi:10.3389/fncel.2019.00531)
Supplement: Supplementary file 1 [file Data_Sheet_1.doc]

**Supplementary Information**

**Small-molecule lysophosphatidic acid receptor 5 (LPAR5) antagonists: Versatile pharmacological tools to regulate inflammatory signaling in BV-2 microglia cells**

**Ioanna Plastira^1^, Lisha Joshi^1^, Eva Bernhart^1^, Jens Schoene^2^, Edgar Specker^2^, Marc Nazare^2, 3^, Wolfgang Sattler^1, 4 *^**

^1^Gottfried Schatz Research Center, Molecular Biology and Biochemistry, Medical University of Graz, Austria;

^2^Leibniz-Forschungsinstitut für Molekulare Pharmakologie (FMP), Berlin, Germany;

^3^Berlin Institute of Health (BIH), Berlin, Germany;

^4^Center for Explorative Lipidomics, BioTechMed-Graz, Austria

**
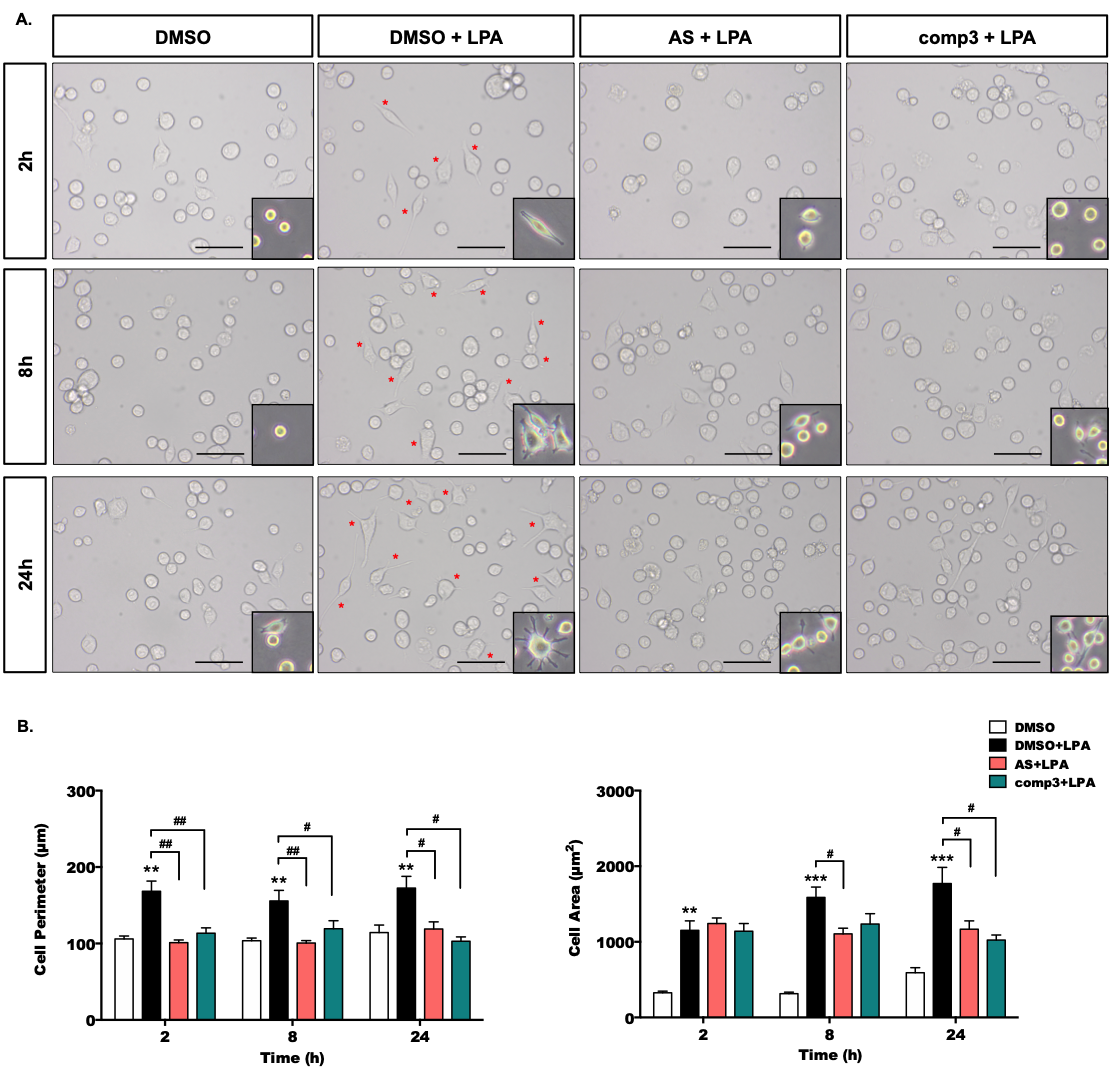
**

**Fig. S1: LPAR5 antagonists attenuate LPA-induced morphological changes**

(**A**) BV-2 cells were cultured in the presence of DMSO or DMSO plus LPA (1 µM) in the absence or presence of AS2717638 (0.1 µM) or compound 3 (1 µM) for the indicated time periods. Representative micrographs (bright field) depict morphological changes upon treatments.

(**B**) Morphological analysis (cell perimeter and area) was performed using ImageJ. At least 50 cells out of 4 different areas per well were measured in two independent experiments. The results are presented as mean + SEM (**p<0.01, ***p<0.001 compared to DMSO-treated cells; ^#^p<0.05; ^##^p<0.01 each inhibitor compared to LPA-treated cells; unpaired Student t-test with Holm-Sidak correction). Scale bars (bright field) = 80 µm. Phase contrast inserts (100x100 pixel) represent magnified images indicating changes in cell morphology under each condition.


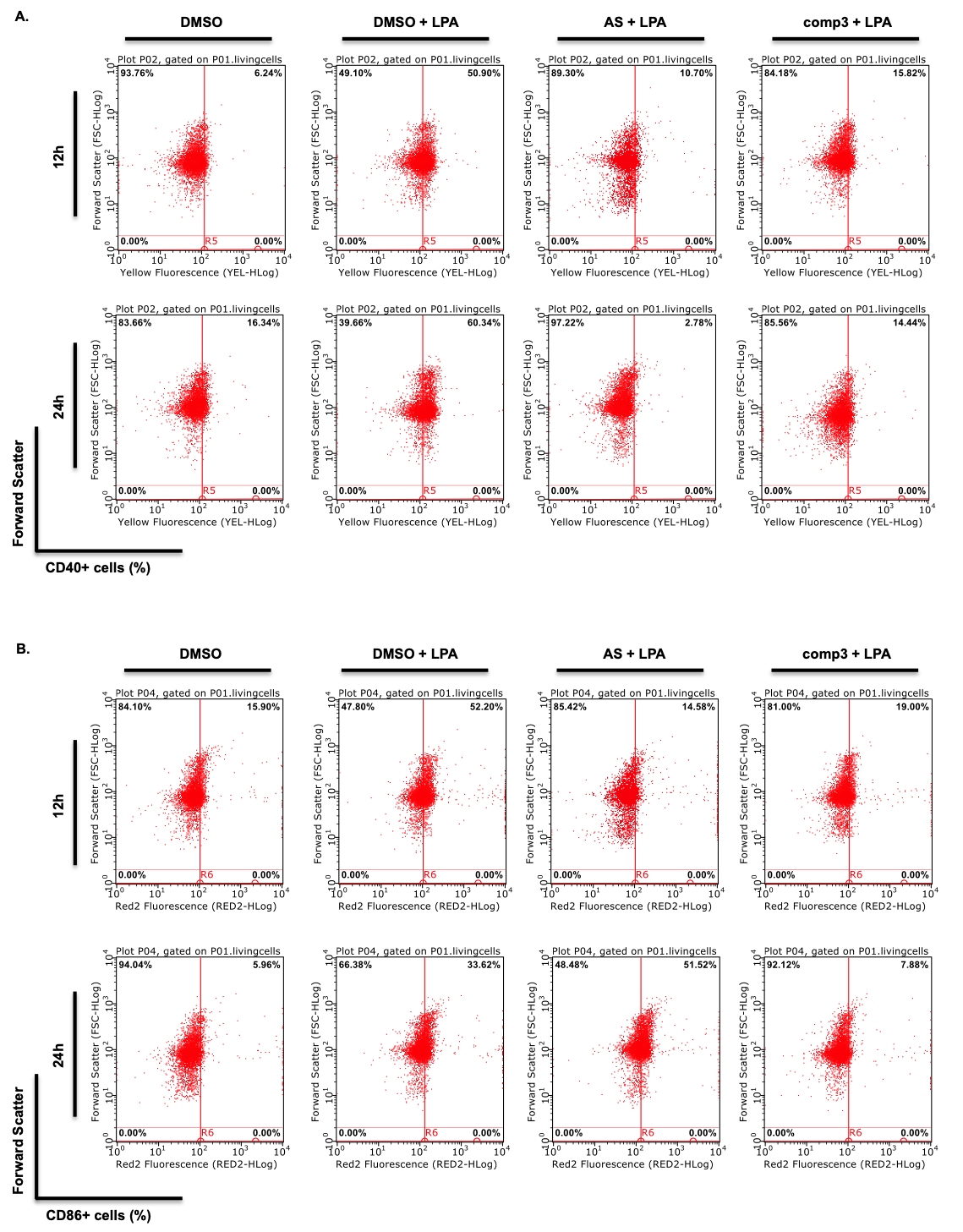


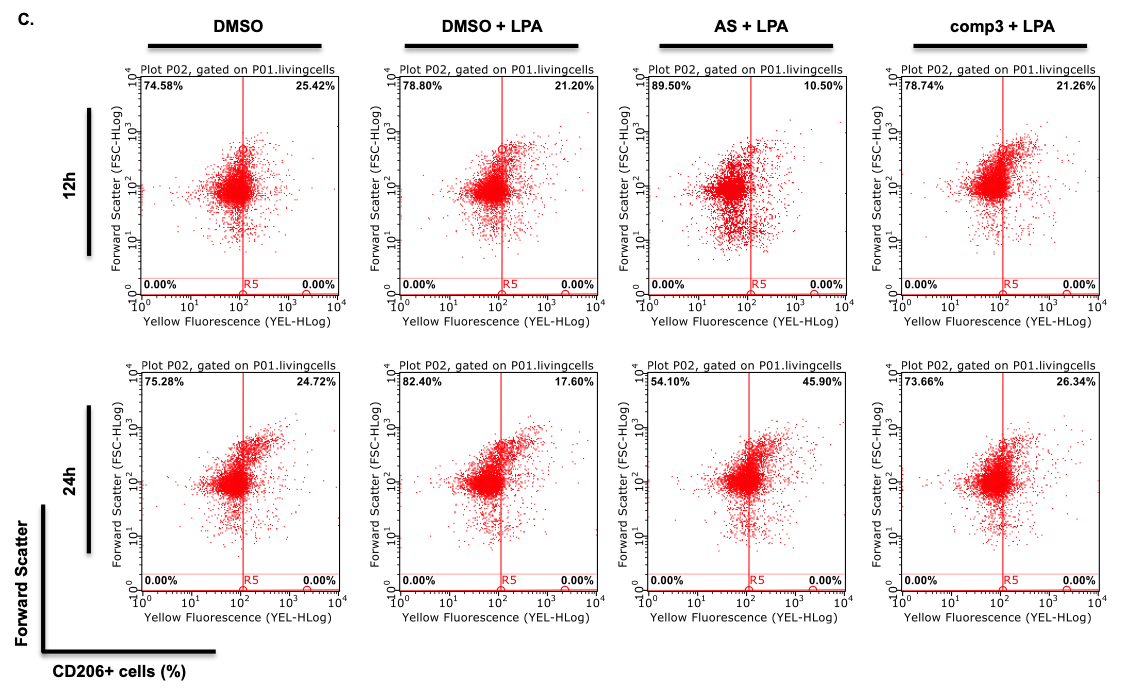


**Fig. S2 (related to figure 4): Representative scatterplots of flow cytometry experiments**

Serum-starved (o/n) BV-2 cells were cultivated in the presence of DMSO or DMSO plus LPA in the absence or presence of AS2717638 (0.1 µM) or compound 3 (1 µM) for the indicated times. Cells were stained with PE-conjugated anti-CD40 (**A**), APC-conjugated anti-CD86 (**B**), or PE-conjugated anti-CD206 (**C**) antibodies and analyzed using a Guava easyCyte 8 Millipore flow cytometer. Representative scatter graphs from one experiment are shown.
